# Insights into the Major Metabolites Involved in the Underground Floral Differentiation of *Erythronium japonicum*

**DOI:** 10.1155/2022/7431151

**Published:** 2022-05-13

**Authors:** Hongtao Wang, Junyi Zhu, Lifan Zhang, Peng Shen, Zi Xiao, Rengui Zhao

**Affiliations:** ^1^School of Life Sciences, Tonghua Teachers College, Tonghua, China; ^2^College of Agriculture, Jilin Agricultural University, Jilin, China

## Abstract

**Background:**

*Erythronium japonicum* Decne (Liliaceae) is an early spring ephemeral with an underground initial floral differentiation stage. The flowering mechanism is crucial in ornamental plants due to the associated economic value. Therefore, this study is aimed at exploring the metabolic landscape during floral differentiation, including flower primordium, perianth, stamen, and the pistil differentiation period, in *E. japonicum* coupled with a conjoint analysis of the metabolome and transcriptome. Using ultraperformance liquid chromatography-tandem mass spectrometry, we identified 586 metabolites from 13 major metabolite classes. Comparative metabolomics between different floral developmental stages revealed several abundant metabolites during the respective phases. Upaccumulation of p-coumaroylputrescine, scopoletin, isorhoifolin, cosmosiin, genistin, and LysoPC 15 : 0 emphasized the significance of these compounds during flower development. Furthermore, previously identified DEGs, viz., *EARLY FLOWERING 3*, *Flowering locus K*, *PHD finger-containing protein*, and *zinc finger SWIM domain-containing protein* for floral differentiation, depicted a high correlation with lipid, flavonoid, and phenolics accumulation during floral developmental stages.

**Conclusions:**

Together, the results improve our interpretation of the underground floral development in *E. japonicum*.

## 1. Introduction

*Erythronium japonicum*, native to Asia, is an early spring ephemeral recently domesticated for its commercial use [1, 2]. Northeast China, Japan, and Korea are considered the geographic origin of *E. japonicum* [1, 3]. The reproductive phase in *E. japonicum* starts underground without vernalization and photoperiod [2]. Flower bud initiation usually starts before dormancy induction in ephemerals plants and continues afterward [4]. Several studies provided significant insights into the growth cycle, propagation, morphological attributes, and environmental impacts in *E. japonicum* [1, 5–9]. However, there is an apparent gap in research addressing the regulatory basis of underground floral differentiation in *E. japonicum*.

Floral organ development is critical for ornamental plants to meet commercial requirements [10, 11]. During the past few decades, floriculture industry has been expanded with the inclusion of wildflowers and their domestication for commercial use [12]. Aside from commercial use, flower development is also critical for plant ecology and evolution [13, 14]. The role of metabolites in pigmentation has been attributed to the variable accumulation of flavonoids, carotenoids, betalains, and chlorophylls [15]. However, metabolic changes associated with active compounds during the floral developmental stage have not been studied well. Metabolomics regulated downstream of genomics and proteomics in system biology provides tools for identifying differentially accumulated metabolites during a specific developmental stage [16]. Several reports suggested distinct metabolic profiles associated with each genotype [17, 18]. Furthermore, differential accumulation of metabolites between different growth stages coupled with transcriptome can provide potential insights into the developmental process.

The shift from vegetative to reproductive stage comprised a set of fine-tuned processes regulated through developmental signals and environmental cues such as light and temperature [19]. The flowering originates from floral primordium followed by stamens and pistil differentiation which are the critical phases in flowers development [20]. Therefore, understanding the regulatory mechanisms underlying stage-specific differentiation is essential. Floral differentiation has gained much attention in plant species. For instance, *Jatropha curcas* [20], *Brassica napus* [21], *Camellia sinensis* [22], *Populus* [23], *Dianthus caryophyllus* [24], *Litsea cubeba* [25], *Rosa chinensi*s [26], *Lilium* [27], and *Juglans regia* [28] have been well studied for molecular mechanisms underlying reproduction phase. Previous statistics suggested the regulatory role of anthocyanins, gibberellic acid, flavonoids, vernalization, and developmental pathways during floral differentiation [29–31]. Florigen, encoding a conserved protein *FLOWERING LOCUS T*, is a systematic switch for flowering control [32]. Moreover, *FT* regulation is mediated by *GIGANTEA–CONSTANS–FT* complex under strict photoperiod sensitivity [22, 32]. The complex regulatory network of flowering is activated by external variables, such as day length and humidity. The environmental variables play a crucial during floral differentiation [33]. However, an overlap between flowering and dormancy regulation pathways complicates understanding specific regulatory pathways [33]. Studies have reported simultaneous reduced expression of *FL* and *FRIGIDA* during vernalization [34, 35]. Understanding the overlap between flowering and dormancy can provide mechanistic insight into flowering regulation during dormancy period.

In this study, we profiled the metabolome and transcriptome of *E. japonicum* at four floral development stages (flower primordium differentiation, perianth differentiation, stamen differentiation, and the pistil differentiation period) to understand the molecular changes underlying the underground floral differentiation.

## 2. Materials and Methods

### 2.1. Plant Material and Sampling

The study area includes the Tuodaoling region in Northeast China, with a high occurrence of *Erythronium japonicum* Decne (Liliaceae) populations. Plant samples were collected to the protocol described in our previous study [36]. Samples were collected in three replicates at four different stages, including flower primordium differentiation, perianth differentiation, stamen differentiation, and the pistil differentiation period. Cryopreserved samples were used for further downstream analysis following the methods of Wang et al. [36] and Gao et al. [37].

### 2.2. UPLC-MS/MS Analysis

*E. japonicum* floral differentiation was characterized using the widely targeted metabolomics approach with ultraperformance liquid chromatography-tandem mass spectrometry (UPLC-MS/MS performed by Metware, Wuhan, China). A series of procedures for metabolite extraction, identification, and quantification were carried out at Wuhan Metware Biotechnology Co., Ltd (https://www.metware.cn), following the company's standard procedures [38, 39]. Cryo-preserved samples were weighed and extracted with 1.0 ml of 70% methanol at 4°C. Extracts were analyzed using liquid chromatography mass-spectrometry/M.S. analysis (LC-MS/MS, UPLC, Shim-pack UFLC SHIMADZU CBM30A system; MS, Applied Biosystems 6500 QTRAP). All metabolites were identified and quantified by Metware's own metabolite database and public metabolite database. Differential accumulation of metabolites (DAMs) between samples was identified using orthogonal partial least squares discriminant analysis. Metabolites with |Log2 Foldchange| ≥ 1 and VIP (variable importance in project) ≥ 1 were defined as DAMs.

### 2.3. Quality Check

Quality check for multistage metabolome was performed according to Fiehn et al. [40]. Descriptive statistics for each dataset were obtained using Analyst 1.6.3 software (AB Sciex, Ontario, ON, Canada). Moreover, variability of datasets was estimated using principal component analysis (PCA) and Pearson correlation coefficient (PCC) with prcomp within R (http://www.r-project.org/).

### 2.4. Identification of Differential Metabolites

Differentially accumulated metabolites (DAMs) between four flower development stages were assessed by exploring the variable importance in projection (VIP) values greater than 1 with Log2 fold change (FC) also greater than 1. Data were transformed to Log_2_FC, and then Orthogonal Projections to Latent Structures-Discriminant Analysis (OPLS-DA) and mean centering were performed. Variable importance in projection (VIP) scores were extracted from OPLS-DA results, with 200 permutations, using R software with MetaboAnalystR package.

### 2.5. Conjoint Analysis of Metabolome and Transcriptome

Metabolome and transcriptome (unpublished and available at PRJNA730644) of four floral differentiation stages were utilized to perform conjoint analysis. Principal component analysis (PCA), KEGG annotation, nine-quadrant, and Pearson correlation analysis between DEGs and DAMs were performed.

## 3. Results

### 3.1. Metabolic Profiling

Metabolites are generally considered as the bridge between molecular mechanisms and phenotype. Therefore, to understand the molecular characteristics underlying flowering in *E. japonicum*, we performed systematic metabolic profiling based on widely targeted metabolomics (UPLC-MS/MS) at four floral differentiation stages, including flower primordium differentiation (Az), perianth differentiation (Bz), stamen differentiation (Cz), and the pistil differentiation period (Dz). The quality was confirmed by monitoring the instrument's accuracy following quality-control measures, as formerly described by Fiehn et al. [40]. Superimposed display analysis of mass spectrometry total ion current (TIC) and extracted-ion chromatogram (XIC) of samples which were run at a different time (Figure S1). The overlapped TIC suggested the stability of the instrument as a quality check.

**Figure 1:**
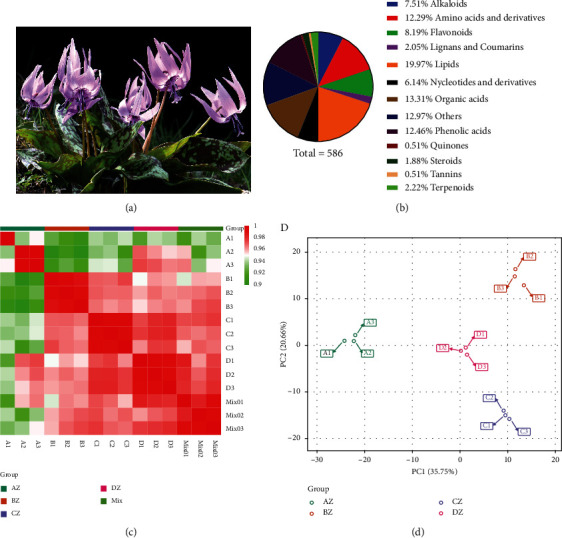
Metabolome quality control and description. (a) Pictorial description of *E. japonicum* plant. (b) Proportion of major identified metabolite classes. (c) Correlation matrix for metabolites identified in flower primordium differentiation (Az), perianth differentiation (Bz), stamen differentiation (Cz), and the pistil differentiation period (Dz). While mix sample was used as a quality check. (d) Principal component analysis for metabolites identified at flower primordium differentiation (Az), perianth differentiation (Bz), stamen differentiation (Cz), and the pistil differentiation period (Dz) in *E. japonicum.*

We identified 586 metabolites (Table S1 and Figure 1) and further categorized these metabolites based on their primary structures into thirteen major classes (Figure 1(b)), including lipids (19.97%), organic acids (13.31%), phenolics (13.31%), amino acids and derivatives (12.29%), flavonoids (8.19%), alkaloids (7.51%), nucleotides and derivatives (6.14%), terpenoids (2.22%), lignans and coumarins (2.05%), steroids (1.88%), tannins (0.51%), quinones (0.51%), and others (12.97%). Complete annotation information concerning identified metabolites has been listed in Table S1.

The results were further verified using PCC and PCA for all the samples (Figures 1(c) and 1(d)). A strong correlation within replicates of each sample was observed, conferring the consistency of metabolome datasets used in the study. Metabolome from different tissues depicted a relatively weak correlation between different samples. Moreover, a scatter plot based on the first two PCs clustered samples into four groups consisting of replicates from each sample. PCA results, depicting 58.41 variations with PC1 (37.75%) and PC2 (20.66%), also verified the reliability of the metabolome dataset.

**Figure 2:**
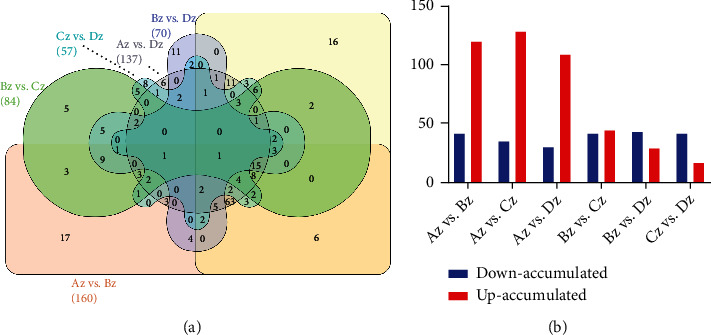
Comparative metabolic profile. (a) Venn diagram representing shared DAMs between different groups. (b) Number of DAMs as upaccumulated and downaccumulated between different floral development stages including flower primordium differentiation (Az), perianth differentiation (Bz), stamen differentiation (Cz), and the pistil differentiation period (Dz) in *E. japonicum.*

We compared the metabolic profiles to clarify the differentially regulated metabolite accumulation between four floral developmental stages (Figure 2). The comparative metabolic profile suggested differential regulation of metabolites between different developmental stages. One hundred sixty-two metabolites were found with differential regulation between primordium differentiation (Az) and perianth differentiation (Bz). Among 160 DAMs, 119 were upaccumulated, and 41 depicted downaccumulation (Table S2). Similarly, 162 (34D and 128 U), 137 (29D and 108 U), 84 (40D and 44 U), 70 (42D and 28 U), and 57 (41D and 16 U) DAMs resulted from the comparison of primordium differentiation (Az) vs. stamen differentiation (Cz), primordium differentiation (Az) vs. pistil differentiation period (Dz), perianth differentiation (Bz) vs. stamen differentiation (Cz), perianth differentiation (Bz) vs. pistil differentiation period (Dz), and stamen differentiation (Cz) vs. pistil differentiation period (Dz), respectively (Figure 2(b), Table S3-S7). The results suggested a significantly higher number of DAMs when the primordium differentiation stage was compared with later stages. However, the number of DAMs reduced when later stages were compared. Furthermore, we identified 100 conserved DAMs when the flower primordium differentiation (Az) stage was compared with perianth differentiation (Bz), stamen differentiation (Cz), and the pistil differentiation period (Dz). KEGG enrichment analysis showed that these pathways are related to the cellular differentiation and transition from vegetative to reproductive stage, suggesting a significant role of DAMs in floral differentiation concerning *E. japonicum.*

**Figure 3:**
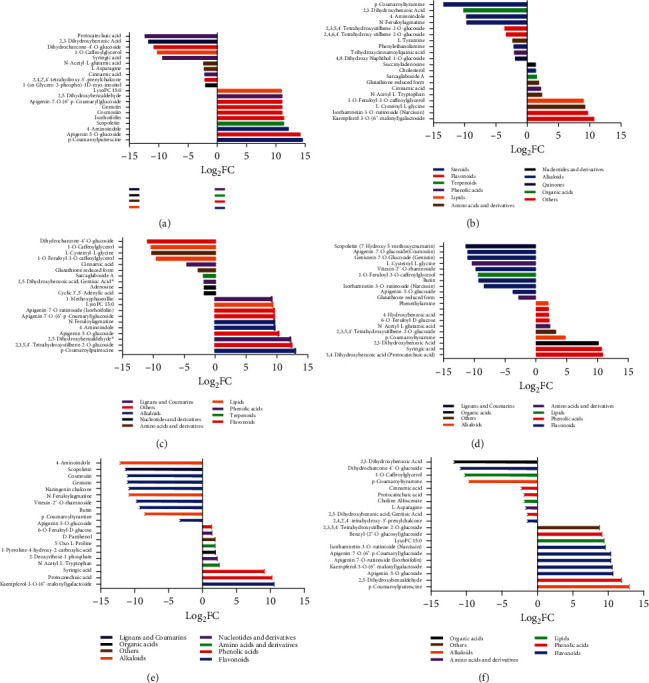
Top fold change (FC) metabolites in comparison of floral developmental stages. (a) Top FC metabolites from flower primordium differentiation (Az) vs. perianth differentiation (Bz). (b) Top FC metabolites from flower primordium differentiation (Az) vs. stamen differentiation (Cz). (c) Top FC metabolites from flower primordium differentiation (Az) vs. pistil differentiation period (Dz). (d) Top FC metabolites from perianth differentiation (Bz) vs. stamen differentiation (Cz). (e) Top FC metabolites from perianth differentiation (Bz) vs. pistil differentiation period (Dz). (f) Top FC metabolites from stamen differentiation (Cz) vs. pistil differentiation period (Dz).

To further understand the differential metabolic landscape at the floral developmental stages, we identified the top 10 metabolites for each extreme (upaccumulated and downaccumulated metabolites; Figure 3, Table S2). Primordium differentiation (Az) and perianth differentiation (Bz) (Az vs. Bz) depicted upaccumulation of p-coumaroylputrescine, apigenin-5-O-glucoside4-aminoindole, scopoletin, isorhoifolin, cosmosiin, genistin, apigenin-7-O-(6”-p-coumaryl)glucoside, 2,5-dihydroxybenzaldehyde, and LysoPC 15 : 0 at perianth differentiation stage. While 1-(sn-glycero-3-phospho)-1D-myo-inositol, 2,4,2',4'-tetrahydroxy-3'-prenylchalcone, cinnamic acid, L-asparagine, N-acetyl-L-glutamic acid, syringic acid, 1-O-caffeoylglycerol, dihydrocharcone-4'-O-glucoside, 2,3-dihydroxybenzoic acid, and protocatechuic acid were downaccumulated at the perianth differentiation stage. Significant enriched KEGG pathways associated with these metabolites included purine metabolism and phenylpropanoid biosynthesis (Figure S2), suggesting significance of these pathways in the early floral differentiation stages.

Comparison of primordium differentiation (Az) and stamen differentiation (Cz) identified kaempferol-3-O-(6”-malonyl)galactoside, isorhamnetin-3-O-rutinoside (narcissin), L-cysteinyl-L-glycine, 1-O-feruloyl-3-O-caffeoylglycerol, N-acetyl-L-tryptophan, cinnamic acid, glutathione reduced form, sarcaglaboside A, cholesterol, and succinyladenosine as significantly upaccumulated at stamen differentiation stage (Figure 3(b)). Similarly, a comparison of primordium differentiation (Az) and pistil differentiation (Dz) suggested significant upaccumulation of p-coumaroylputrescine, 2,3,5,4'-tetrahydroxystilbene-2-O-glucoside, 4-aminoindole, LysoPC 15 : 0, 2,5-dihydroxybenzaldehyde, apigenin-5-O-glucoside, N-feruloylagmatine, apigenin-7-O-(6”-p-coumaryl)glucoside, apigenin-7-O-rutinoside (isorhoifolin), and 1-methoxyphaseollin (Figure 3(c)). KEGG enrichment analysis for the identified DAMs suggested an association with purine metabolism, phenylalanine metabolism, propanoate metabolism, and photosynthesis (Figure S3). Comparisons of later stages (Bz vs. Cz (Figure 3(d)), Bz vs. Dz (Figure 3(e)), and Cz vs. Dz (Figure 3(f))) showed significant differential regulation of flavonoids, lignans, coumarins, phenolics, and alkaloids. KEGG enrichment analysis illustrated a significant association of DAMs with phenylalanine biosynthesis, glutathione biosynthesis, and glucosinolate biosynthesis.

### 3.2. Conjoint Analysis of Metabolome and Transcriptome

Metabolome data sets of *E. japonicum* were further exploited using conjoint analysis of metabolic and transcriptomic profiles of multistage floral development, including flower primordium differentiation (AZ), perianth differentiation (BZ), stamen differentiation (CZ), and the pistil differentiation period (DZ). The transcriptome data sets used in this study resulted in the identification of 9,383, 6,979, 16,758, 9,522, 7,387, and 12,502 DEGs in Az vs. Bz, Az vs. Cz, Az vs. Dz, Bz vs. Cz, Bz vs. Dz, and Cz vs. Dz, respectively [36]. Furthermore, 48, 33, 59, 42, 34, and 54 DEGs were identified related to floral differentiation in Az vs. Bz, Az vs. Cz, Az vs. Dz, Bz vs. Cz, Bz vs. Dz, and Cz vs. Dz, respectively. To further confirm the relationship between transcriptome and metabolome of respective tissue, conjoint analysis was performed. The KEGG enrichment analysis for all DEGs and DAMs was performed for each comparison.

**Figure 4:**
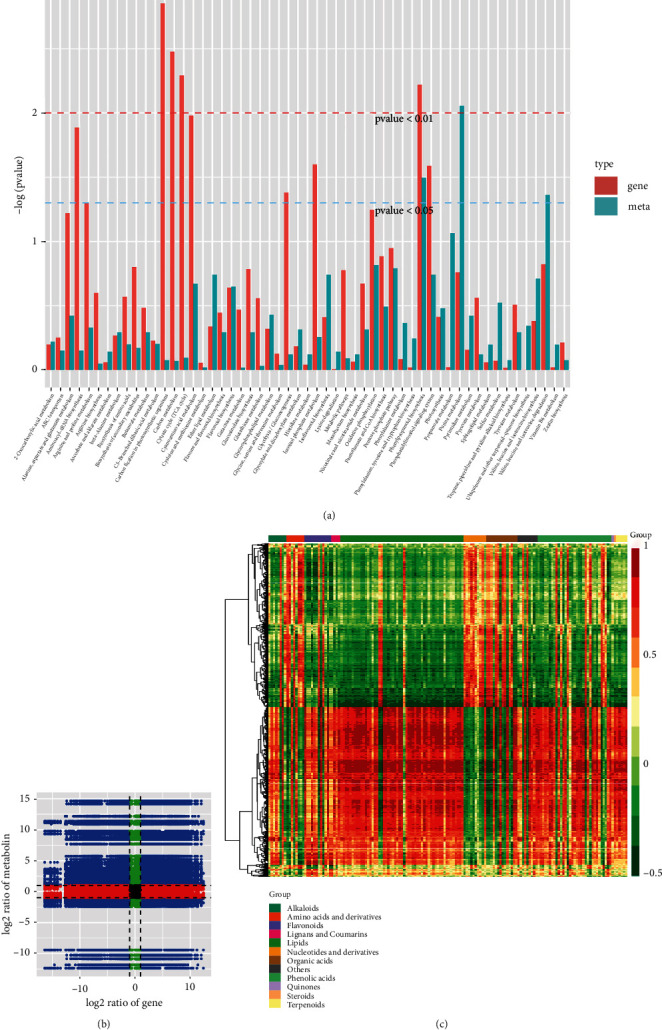
Conjoint analysis of DAMs and DEGs in flower primordium differentiation (Az) vs. perianth differentiation (Bz). (a) KEGG enrichment for DEGs and DAMs. (b) 9-Quadrant graph representing DAMs and DEGs with correlation (PCC) higher than 0.8. (c) Heatmap representing DMAs with PCC higher than 0.8.

As presented in Figure 4, comparative transcriptome and metabolome between flower primordium differentiation (AZ) and perianth differentiation (BZ) stage suggested KEGG enrichment of DEGs and DAMs in multiple regulatory pathways, including flavonoid biosynthesis, photosynthesis, and lipid metabolism (Figure 4(a)). The PCC for DAMs and DEGs was calculated using R and PCC with values higher than 0.8 are presented as a nine-quadrant diagram (Figure 4(b)). Quadrants 3 and 7 represented the DEGs and DAMs with consistent regulation (positively correlated). In contrast, quadrants 1, 2, 4, 6, 8, and 9 represented DEGs and DAMs with negative correlations. DEGs and DAMs with PCC greater than 0.8 were selected, and their expression pattern was presented as a heat map (Figure 4(c)). The clustered heat map depicted DAMs highly correlated with DEGs into 12 major classes, with the most abundant classes as flavonoids, lipids, and phenolics.

**Figure 5:**
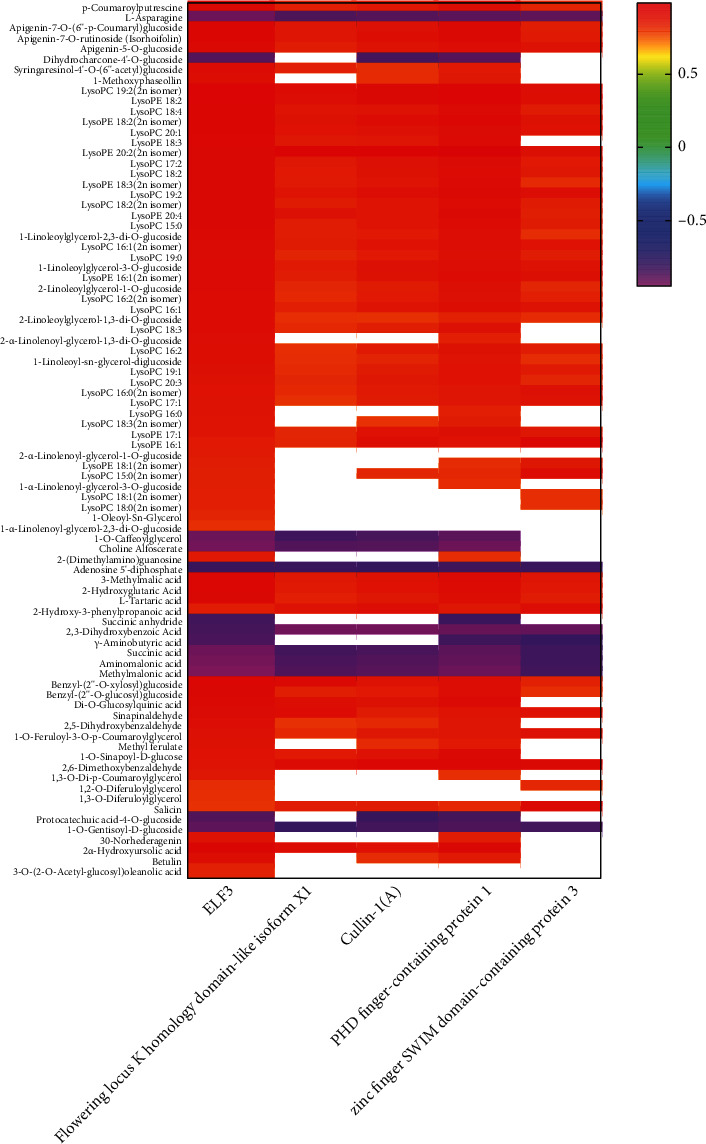
Correlation of five selected DEGs with DAMs identified from comparison Az vs. Bz.

Correlation analysis of *Cluster-35905.71088*; *EARLY FLOWERING 3* (*ELF 3*) with DAMs identified at the perianth differentiation stage suggested a significantly higher correlation between *ELF 3* and DAMs (Figure 5). Lipids were among the most abundant metabolite class significantly correlated with *ELF3*, suggesting a substantial role of lipids in flower morphogenesis. All lipids showed a positive correlation with *ELF3* except 1-O-caffeoylglycerol and choline alfoscerate, which showed a significant negative correlation with *ELF 3*. Three flavonoids, including apigenin-7-O-(6”-p-coumaryl)glucoside, apigenin-7-O-rutinoside (isorhoifolin), and apigenin-5-O-glucoside depicted a positive correlation with *ELF 3*, while dihydrocharcone-4'-O-glucoside was found with a significant negative correlation. Among 15 phenolics, 13 expressed a positive correlation with *ELF 3*. *Flowering locus K*, *cullin-1*, *PHD finger-containing protein*, and *ZSWIM3* (*zinc finger SWIM domain-containing protein 3*) also positively correlated with lipid accumulation.

## 4. Discussion

Flower development is a systematic process under strict genetic control in higher plants. It can be divided into major phases: floral induction, meristem formation, and floral organ development [41]. The complex flowering mechanism is controlled by highly conserved genes regulating transcription factors and their protein products composing a gene regulatory network (GRN). Flowering-time genes are at the top of GRN hierarchy and play a crucial role in the developmental shift. Flowering control genes are generally triggered by external factors such as photoperiod, temperature, and humidity [42, 43]. However, the initial flowering phase in *E. japonicum* starts underground in the absence of light and vernalization [2]. Therefore, it is valuable to explore and identify the genetic regulators of floral differentiation in *E. japonicum*. The present study aimed at providing a systematic metabolic insight coupled with a conjoint analysis of the metabolome and transcriptome at four floral developmental stages in *E. japonicum* viz., primordium differentiation, perianth differentiation, stamen differentiation, and pistil differentiation stage.

The results suggested significant differences in metabolite accumulation at different developmental stages identifying 586 differentially accumulated metabolites (DAM). The specified DAMs were classified into thirteen major classes. A similar approach has been adapted in multiple species to utilize omics-approach in identifying regulatory mechanisms of flowering such as *Cannabis sativa* [44], *Lonicera japonica* [45], *Brassica juncea* [46], *Ranunculus glacialis* [47], *Chrysanthemum morifolium* [48], *Chrysanthemum lavandulifolium* [49], *Staphisagria Ranunculaceae* [50], and Delphinieae [51].

Flowering time is important in securing seed production and, therefore, ensuring species survival. In addition to the gene regulatory network controlling floral differentiation, there is a significant lack of studies concerning the role of metabolites during flower development stages. Multistage metabolome comparison yielded significant variation in metabolite accumulation. For instance, a comparison of primordium differentiation (Az) and perianth differentiation (Bz) stages identified upaccumulation of p-coumaroylputrescine, scopoletin, isorhoifolin, cosmosiin, genistin, and LysoPC 15 : 0. p-Coumaroylputrescine has been previously identified with a substantial role during flower development in tobacco [52–54]. Scopoletin plays a significant role in physiological activities [55]. Therefore upaccumulation of scopoletin and its glycosides during the perianth differentiation suggested a considerable role of scopoletin in transition from primordium differentiation to perianth differentiation. Although cosmosiin, genistin, and LysoPC 15 : 0 have been identified at the flowering stage in multiple species, their roles during the floral differentiation stages are still unknown. Furthermore, gene ontology annotation for the DMAs during the perianth differentiation stage suggested enrichment of purine metabolism and phenylpropanoid biosynthesis. The phenylpropanoid biosynthetic pathway is crucial for flavonoid pigments synthesis during flower development [56, 57].

A multifaceted gene regulatory network constituting a hierarchy of coordinated gene functions is crucial for flower development. Most of the genes involved in the floral gene regulatory network encode transcription factors, such as MADS-domain, LEAFY- (LFY-) like, and APETALA2- (AP2-) like proteins [41]. Transcriptome analysis of four floral differentiation stages of *E. japonicum* suggested a differential expression of *ELF3* and *FT*, *cullin 1*, *GLP1*, and *CONSTANS* [36]. We performed the conjoint analysis of metabolome and transcriptome to understand the coregulation of DEGs and DAMs. ELF3 expression was significantly correlated with lipids and flavonoids. The ELF3 gene, also known as the clock gene, regulates the evening complex (EC) with ELF4 and LUX leading to flowering [58, 59]. Furthermore, Deng et al. characterized CONSTANS gene-regulating lipid biosynthesis during flower development in *Chlamydomonas reinhardtii* [60]. *Flowering locus K*, *cullin-1*, *PHD finger-containing protein*, and *ZSWIM3* (*zinc finger SWIM domain-containing protein 3*) also depicted a positive correlation with lipid accumulation. Further molecular characterization of identified DEGs and DAMs can yield potential insights into gene regulatory network of flowering and metabolite regulation during floral development.

In sum, this study utilized the metabolome and transcriptome of *E. japonicum* at four underground flower developmental stages. Comparative metabolomics suggested a differential accumulation of 586 metabolites during floral differentiation. The identified DAMs were further narrowed down based on their accumulation pattern. A conjoint analysis of metabolome and transcriptome yielded insight into coregulation of DEGs and DAMs, suggesting a close link between regulation of flowering-related genes and metabolite accumulation. Further molecular insights can potentially highlight the role of metabolites during floral differentiation in *E. japonicum.*

## Data Availability

The raw RNA-seq data have been submitted to NCBI SRA under the project number: PRJNA730644.

## Supplementary Materials

Supplementary Materials Figure S1: quality check for randomly selected sample. (a, b) Integral correlation chart of quantitative metabolite analysis. The x-axis represents retention time, the y-axis represents ion current intensity (PS), and peak represent relative content of the metabolite. (c) Total ion current (TIC) over time for sample 20. Figure S2: KEGG enrichment analysis. (a) KEGG enrichment for Az vs. Bz. (b) KEGG enrichment for Az vs. Cz. (c) KEGG enrichment for Az vs. Dz. Figure S3: KEGG enrichment analysis. (a) KEGG enrichment for Bz vs. Cz. (b) KEGG enrichment for Bz vs. Dz. (c) KEGG enrichment for Cz vs. Dz. Table S1: metabolic profile of E. japonicum floral developmental stages. Table S2: differentially accumulated metabolites (DAMs) between flower primordium differentiation (Az) and perianth differentiation stage (Bz). Table S3: differentially accumulated metabolites (DAMs) between flower primordium differentiation (Az) and stamen differentiation (Cz). Table S4: differentially accumulated metabolites (DAMs) between flower primordium differentiation (Az) and pistil differentiation period (Dz). Table S5: differentially accumulated metabolites (DAMs) between perianth differentiation stage (Bz) and stamen differentiation (Cz). Table S6: differentially accumulated metabolites (DAMs) between perianth differentiation stage (Bz) and pistil differentiation period (Dz). Table S7: differentially accumulated metabolites (DAMs) between stamen differentiation (Cz) and pistil differentiation period (Dz). Table S8: correlation of five selected DEGs (related to floral differentiation) with DAMs identified from comparison Az vs. Bz.
