# Supplementary figures and images for: Insights into the Major Metabolites Involved in the Underground Floral Differentiation of Erythronium japonicum
Source: Biomed Res Int. 2022 May 13;2022:7431151. doi: 10.1155/2022/7431151 (PMC9122723; doi:10.1155/2022/7431151)

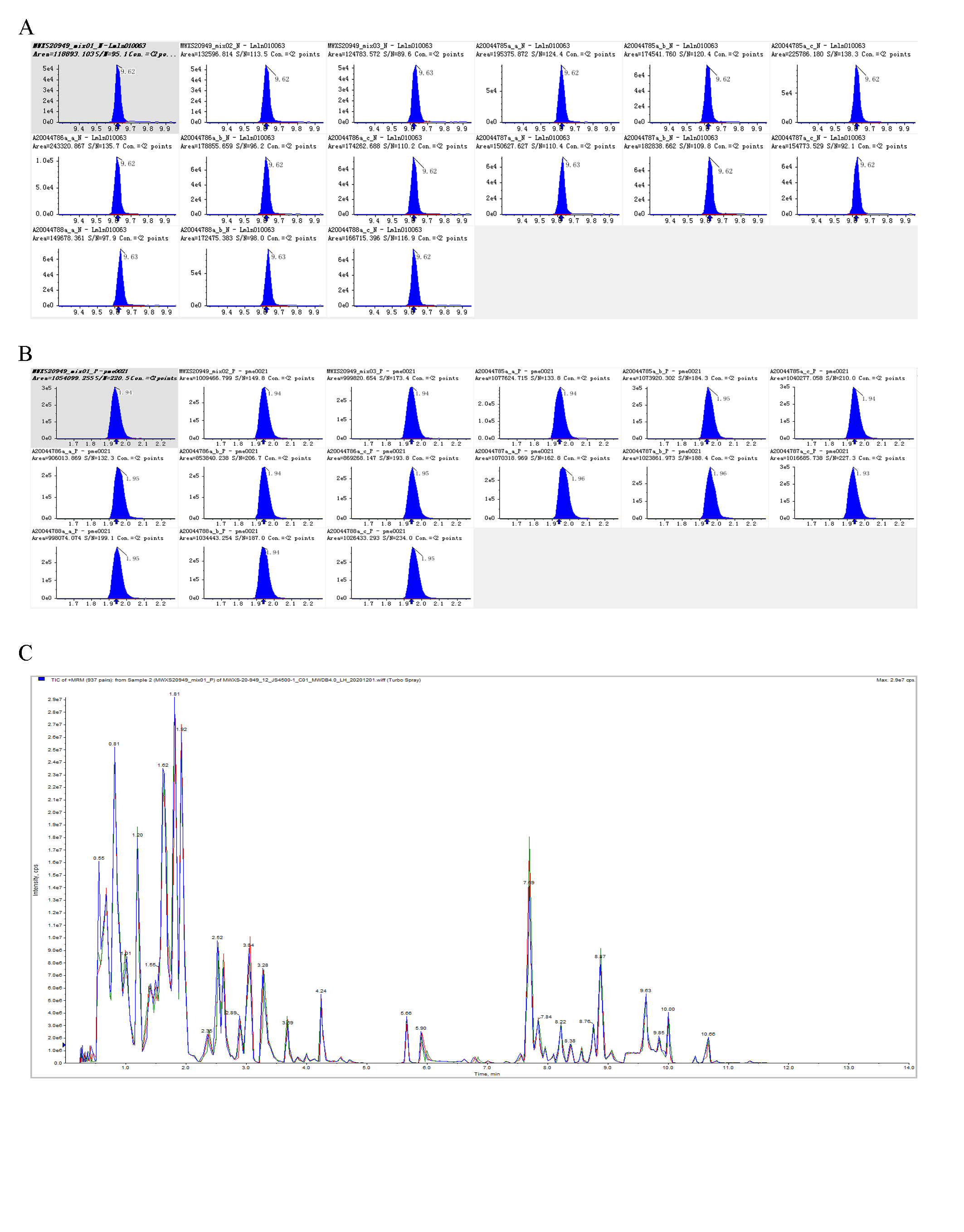

Supplement: Supplementary Materials — Figure S1: quality check for randomly selected sample. (a, b) Integral correlation chart of quantitative metabolite analysis. The x-axis represents retention time, the y-axis represents ion current intensity (PS), and peak represent relative content of the metabolite. (c) Total ion current (TIC) over time for sample 20. Figure S2: KEGG enrichment analysis. (a) KEGG enrichment for Az vs. Bz. (b) KEGG enrichment for Az vs. Cz. (c) KEGG enrichment for Az vs. Dz. Figure S3: KEGG enrichment analysis. (a) KEGG enrichment for Bz vs. Cz. (b) KEGG enrichment for Bz vs. Dz. (c) KEGG enrichment for Cz vs. Dz. Table S1: metabolic profile of E. japonicum floral developmental stages. Table S2: differentially accumulated metabolites (DAMs) between flower primordium differentiation (Az) and perianth differentiation stage (Bz). Table S3: differentially accumulated metabolites (DAMs) between flower primordium differentiation (Az) and stamen differentiation (Cz). Table S4: differentially accumulated metabolites (DAMs) between flower primordium differentiation (Az) and pistil differentiation period (Dz). Table S5: differentially accumulated metabolites (DAMs) between perianth differentiation stage (Bz) and stamen differentiation (Cz). Table S6: differentially accumulated metabolites (DAMs) between perianth differentiation stage (Bz) and pistil differentiation period (Dz). Table S7: differentially accumulated metabolites (DAMs) between stamen differentiation (Cz) and pistil differentiation period (Dz). Table S8: correlation of five selected DEGs (related to floral differentiation) with DAMs identified from comparison Az vs. Bz. [file 7431151.f1.zip › 7431151.f1.jpg]

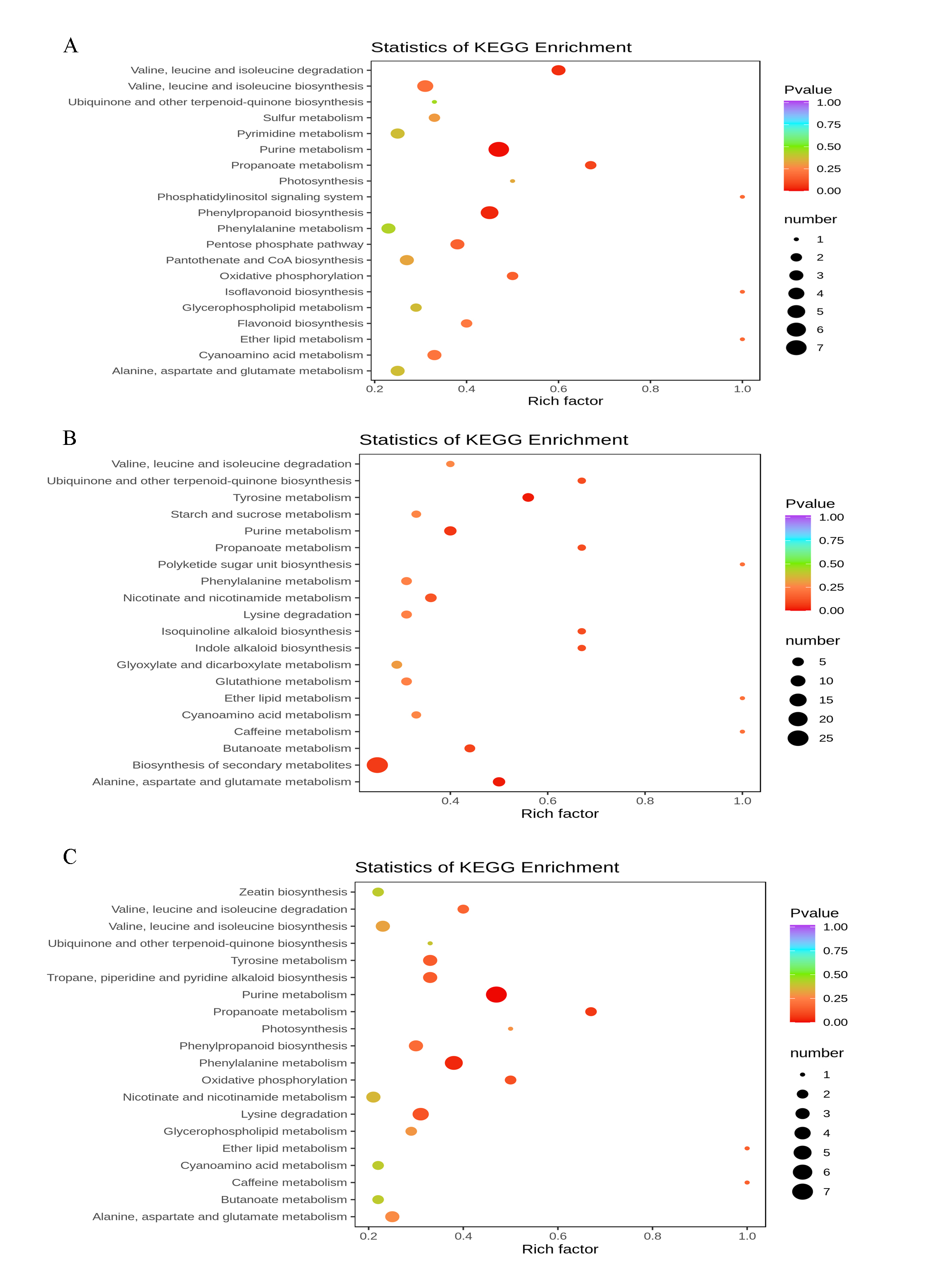

Supplement: Supplementary Materials — Figure S1: quality check for randomly selected sample. (a, b) Integral correlation chart of quantitative metabolite analysis. The x-axis represents retention time, the y-axis represents ion current intensity (PS), and peak represent relative content of the metabolite. (c) Total ion current (TIC) over time for sample 20. Figure S2: KEGG enrichment analysis. (a) KEGG enrichment for Az vs. Bz. (b) KEGG enrichment for Az vs. Cz. (c) KEGG enrichment for Az vs. Dz. Figure S3: KEGG enrichment analysis. (a) KEGG enrichment for Bz vs. Cz. (b) KEGG enrichment for Bz vs. Dz. (c) KEGG enrichment for Cz vs. Dz. Table S1: metabolic profile of E. japonicum floral developmental stages. Table S2: differentially accumulated metabolites (DAMs) between flower primordium differentiation (Az) and perianth differentiation stage (Bz). Table S3: differentially accumulated metabolites (DAMs) between flower primordium differentiation (Az) and stamen differentiation (Cz). Table S4: differentially accumulated metabolites (DAMs) between flower primordium differentiation (Az) and pistil differentiation period (Dz). Table S5: differentially accumulated metabolites (DAMs) between perianth differentiation stage (Bz) and stamen differentiation (Cz). Table S6: differentially accumulated metabolites (DAMs) between perianth differentiation stage (Bz) and pistil differentiation period (Dz). Table S7: differentially accumulated metabolites (DAMs) between stamen differentiation (Cz) and pistil differentiation period (Dz). Table S8: correlation of five selected DEGs (related to floral differentiation) with DAMs identified from comparison Az vs. Bz. [file 7431151.f1.zip › 7431151.f2.jpg]

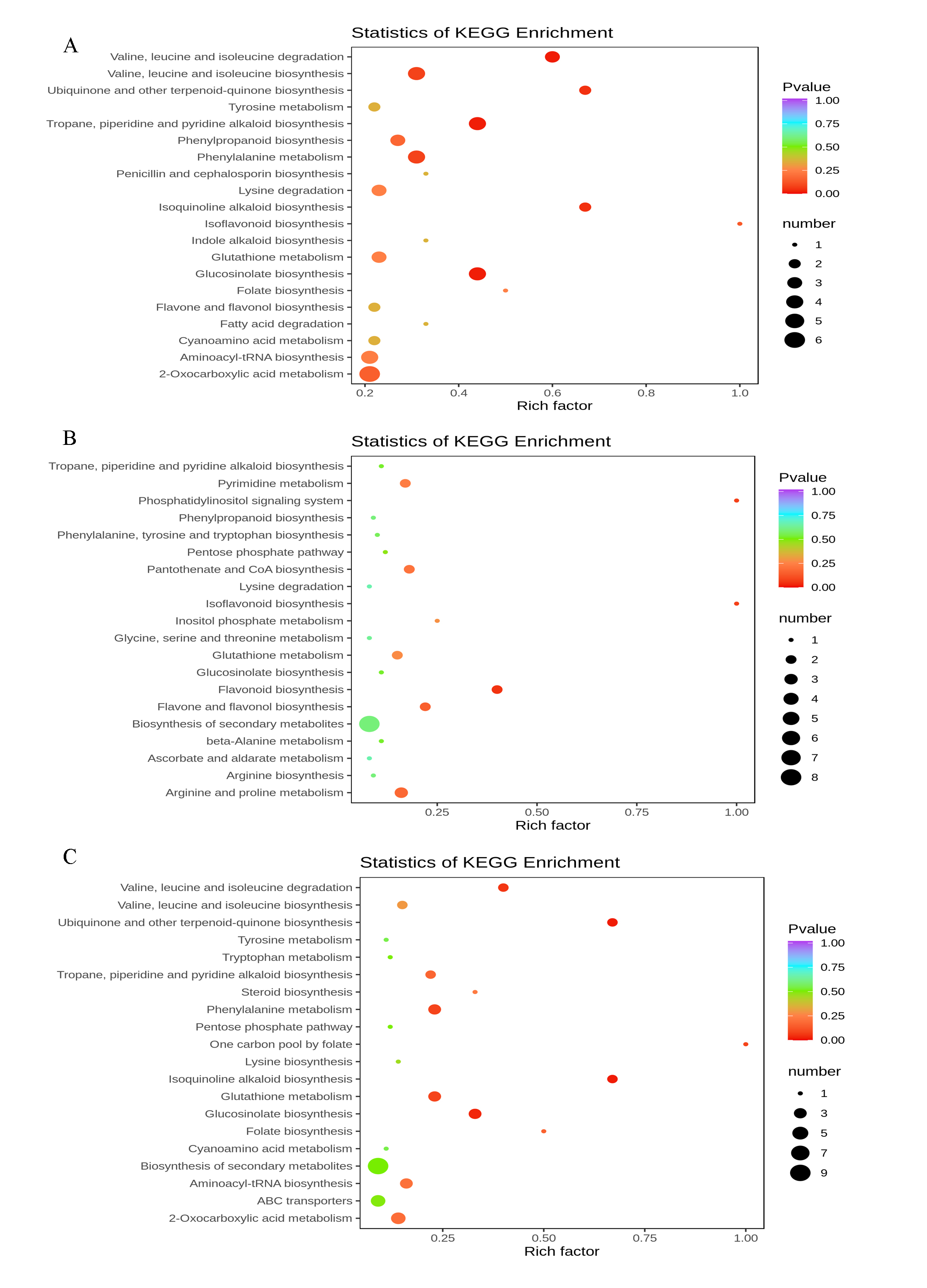

Supplement: Supplementary Materials — Figure S1: quality check for randomly selected sample. (a, b) Integral correlation chart of quantitative metabolite analysis. The x-axis represents retention time, the y-axis represents ion current intensity (PS), and peak represent relative content of the metabolite. (c) Total ion current (TIC) over time for sample 20. Figure S2: KEGG enrichment analysis. (a) KEGG enrichment for Az vs. Bz. (b) KEGG enrichment for Az vs. Cz. (c) KEGG enrichment for Az vs. Dz. Figure S3: KEGG enrichment analysis. (a) KEGG enrichment for Bz vs. Cz. (b) KEGG enrichment for Bz vs. Dz. (c) KEGG enrichment for Cz vs. Dz. Table S1: metabolic profile of E. japonicum floral developmental stages. Table S2: differentially accumulated metabolites (DAMs) between flower primordium differentiation (Az) and perianth differentiation stage (Bz). Table S3: differentially accumulated metabolites (DAMs) between flower primordium differentiation (Az) and stamen differentiation (Cz). Table S4: differentially accumulated metabolites (DAMs) between flower primordium differentiation (Az) and pistil differentiation period (Dz). Table S5: differentially accumulated metabolites (DAMs) between perianth differentiation stage (Bz) and stamen differentiation (Cz). Table S6: differentially accumulated metabolites (DAMs) between perianth differentiation stage (Bz) and pistil differentiation period (Dz). Table S7: differentially accumulated metabolites (DAMs) between stamen differentiation (Cz) and pistil differentiation period (Dz). Table S8: correlation of five selected DEGs (related to floral differentiation) with DAMs identified from comparison Az vs. Bz. [file 7431151.f1.zip › 7431151.f3.jpg]
